# Signaling through the S1P−S1PR Axis in the Gut, the Immune and the Central Nervous System in Multiple Sclerosis: Implication for Pathogenesis and Treatment

**DOI:** 10.3390/cells10113217

**Published:** 2021-11-18

**Authors:** Simela Chatzikonstantinou, Vasiliki Poulidou, Marianthi Arnaoutoglou, Dimitrios Kazis, Ioannis Heliopoulos, Nikolaos Grigoriadis, Marina Boziki

**Affiliations:** 1 3rd Department of Neurology, Aristotle University of Thessaloniki, “G.Papanikolaou” Hospital, Leoforos Papanikolaou, Exohi, 57010 Thessaloniki, Greece; melina.chatzik@gmail.com (S.C.); dimitrioskazis@auth.gr (D.K.); 2 1st Department of Neurology, Aristotle University of Thessaloniki, AHEPA Hospital, 1, Stilp Kyriakidi st., 54636 Thessaloniki, Greece; basia_poulidou@yahoo.gr (V.P.); marnaout@auth.gr (M.A.); 3 Department of Neurology, University General Hospital of Alexandroupolis, Democritus University of Thrace, 68100 Alexandroupoli, Greece; iiliop@med.duth.gr; 4 Multiple Sclerosis Center, Laboratory of Experimental Neurology and Neuroimmunology, 2nd Department of Neurology, Aristotle University of Thessaloniki, AHEPA Hospital, 1, Stilp Kyriakidi st., 54636 Thessaloniki, Greece; ngrigoriaids@auth.gr

**Keywords:** sphingosine 1-phoshate, sphingosine 1-phosphate receptors, multiple sclerosis, gut–brain axis, gut microbiota, fingolimod

## Abstract

Sphingosine 1-phosphate (S1P) is a signaling molecule with complex biological functions that are exerted through the activation of sphingosine 1-phosphate receptors 1–5 (S1PR1–5). S1PR expression is necessary for cell proliferation, angiogenesis, neurogenesis and, importantly, for the egress of lymphocytes from secondary lymphoid organs. Since the inflammatory process is a key element of immune-mediated diseases, including multiple sclerosis (MS), S1PR modulators are currently used to ameliorate systemic immune responses. The ubiquitous expression of S1PRs by immune, intestinal and neural cells has significant implications for the regulation of the gut–brain axis. The dysfunction of this bidirectional communication system may be a significant factor contributing to MS pathogenesis, since an impaired intestinal barrier could lead to interaction between immune cells and microbiota with a potential to initiate abnormal local and systemic immune responses towards the central nervous system (CNS). It appears that the secondary mechanisms of S1PR modulators affecting the gut immune system, the intestinal barrier and directly the CNS, are coordinated to promote therapeutic effects. The scope of this review is to focus on S1P−S1PR functions in the cells of the CNS, the gut and the immune system with particular emphasis on the immunologic effects of S1PR modulation and its implication in MS.

## 1. Introduction

**Figure 1:**
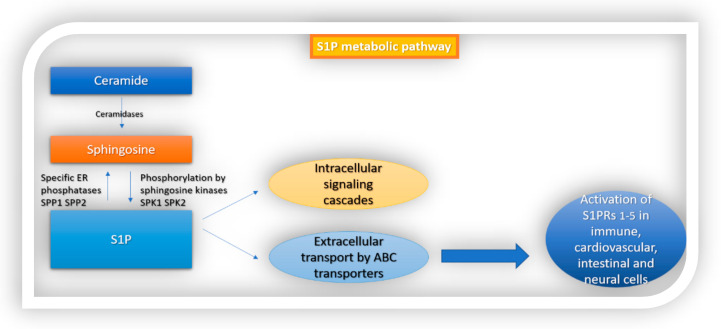
S1P metabolic pathway. S1P is formed from ceramide. Ceramidase converts ceramide to sphingosine, which is then phosphorylated by sphingosine kinases 1 and 2 to S1P. Following its production, S1P interacts with intracellular targets or is transported extracellularly by ABC transporters. Finally, it activates sphingosine 1-phosphate receptors 1–5 (S1PRs _1–5_) which are ubiquitously expressed in different cell types in the body.

Sphingolipids, including sphingomyelin and its metabolites, are structural components of all cell membranes and of the myelin sheath in the nervous system. They were discovered by J.Thudichum in 1874 [1] but it was not until 1997, when the specific, high-affinity G-protein-coupled receptors for the sphingomyelin metabolite sphingosine 1-phosphate (S1P) were identified [2,3], that their multiple physiologic roles in the human body started to be recognized. Subsequent molecular and physiologic research studies suggested that the S1P system mediates various intracellular signaling cascades in the CNS, cardiovascular and immune systems [4]. S1P is a bioactive lipid second messenger that has important signaling functions in cell growth, cell proliferation and angiogenesis. It also regulates biological functions in health and disease [5,6]. S1P concentrations are low in intracellular and interstitial fluids and increased within blood and lymph, in the sub-micromolar range, therefore creating a S1P gradient, which is important for regulating physiologic actions such as lymphocyte egress from secondary lymphoid organs. Multiple enzymes are essential for maintaining the S1P gradient between tissues and systemic circulation and a dynamic equilibrium between S1P and sphingosine. S1P is a lipid metabolite of ceramide. Ceramide can be either broken down by ceramidases to sphingosine, or phosphorylated in the Golgi apparatus by ceramide kinase to produce ceramide-1-phosphate (C1P), another sphingolipid metabolite. In contrast with ceramide and sphingosine, which are considered to activate an apoptotic response, S1P is associated with the suppression of apoptosis [7]. S1P is produced intracellularly through the phosphorylation of sphingosine by sphingosine kinases (SphK1, SphK2), or extracellularly through the hydrolysis of sphingosyl phosphorylcholine by autotoxin. SphK1 is mainly responsible for the cytosolic and extracellular S1P, has trophic functions and can be upregulated in response to proinflammatory cytokines. In contrast, SphK2 can translocate into the nucleus and has the capacity to enhance apoptosis [5]. Following its production, S1P interacts with intracellular targets or is transported extracellularly by ABC transporters, in autocrine or paracrine cell targets, to activate the G-protein-coupled receptor named sphingosine 1-phosphate receptors 1–5 (S1PRs _1–5_) (Figure 1). S1P is converted to sphingosine by S1P-specific ER phosphatases (SPP1 and SPP2) or degraded by S1P lyase (S1PL) [5,8,9].

The main cell sources of circulating blood S1P (~l μM) under normal conditions are erythrocytes and vascular endothelial cells, whereas lymphatic fluid S1P (~0,1 μM) is produced by lymphatic endothelial cells [7,10]. During inflammation and platelet activation, S1P overproduction is due to mast cells’ and platelets’ activation, respectively. In plasma, S1P is mainly bound to high-density lipoprotein (HDL, 60%) and other plasma proteins, such as albumin (30%) [4]. Most HDL-bound S1P is also connected to Apolipoprotein M (ApoM), a S1P chaperon that serves multiple functions. First, it prevents S1P from degradation and enhances interaction with receptors [11]. It also inhibits lymphopoiesis through S1PR1 signaling in bone marrow lymphocyte progenitors [7] and additionally, it suppresses cytokine-induced inflammatory responses in endothelial cells, thus maintaining vascular integrity [11,12]. S1PRs are ubiquitously expressed in the body, including by immune, cardiovascular, intestinal and neural cells [13]. They mediate diverse cellular functions via intracellular signaling cascades, coupling with different G-proteins like Gi, Gq, Go, G12/13 and Rho that activate adenylyl cyclase, PLC, phospholipases D (PLD), ERK, Akt, PI3K, c-Jun N-terminal kinase (JNK), p38 MAPK and non-receptor tyrosine kinases. S1PR1-3 are distributed in most cell types, with high density in cardiovascular and immune systems. S1PR4 and S1PR5 are less numerous and less widely expressed, mainly by lymphatic and nervous cells [11,14].

## 2. S1P Signaling in the Immune System

S1P signaling plays a multifactorial role in immunity. Initially recognized and well-described is the role of S1P−S1PR1 modulation of T-cell trafficking, which has a crucial impact on adaptive immunity [15]. Recent findings suggest, however, additional roles of S1P signaling in B-cell trafficking [7] and also in innate immunity [16]. S1PR1 is widely expressed by T cells, B cells, macrophages, neutrophils, dendritic cells, monocytes, eosinophils, mast cells and NK cells. S1PR2 is expressed on macrophages, monocytes, and eosinophils and mast cells. S1PR3 is expressed on macrophages, neutrophils during inflammation, dendritic cells, monocytes, eosinophils and mast cells. S1PR4 is expressed on macrophages, neutrophils, dendritic cells, monocytes, eosinophils and mast cells. S1PR5 is expressed on patrolling monocytes and NK cells [16,17]. 

### 2.1. Lymphocyte Trafficking and Regulation of Adaptive Immune Functions

S1PR1 expression is required for B and T cells to egress from lymph nodes, for the exit of mature T cells from the thymus and for the migration of natural killer T cells from secondary lymphoid organs into the circulation [18]. The circulation of naïve B and T lymphocytes between the blood and lymphatic systems is important for various functions of the immune system and is orchestrated by S1P and S1PR1.

Described above, the positive S1P gradient between lymphoid organs and the lymphatic circulation is utilized by T lymphocytes to egress from thymus and peripheral lymphoid organs into the lymphatic circulation [7,11]. However, the entry into secondary lymphoid organs does not require S1P signaling. Studies of S1PR1 knockout mice in hematopoietic cells reported significant lymphopenia, resulting from intact lymphocyte maturation but the inability of the last to egress from thymus and peripheral lymphoid organs [19]. Similarly, the inhibition of sphingosine kinase 1/2 or S1P lyase results in lymphopenia, by a similar mechanism [11,20].

In circulation, where S1P concentration is high, T lymphocytes’ S1PR1s are mostly internalized. This desensitization of S1PR1 is a crucial step for circulating T cells to enter lymphoid organs against the S1P gradient. Lymphocytes that cannot achieve the internalization step, due to a lack of GRK2 (a critical regulator of the internalization step) lack the ability to enter lymphoid organs [20]. Upon entrance into the lymphoid organs, where the S1P concentration is low, T cells upregulate S1PR1 surface expression [11]. Activated lymphocytes need to be temporarily retained in the lymph node which is achieved by downmodulation of S1PR1 following the inhibition of KLF2 and upregulation of CD69. CCR7, a chemokine receptor expressed in naive T cells, retains lymphocytes within secondary lymphoid organs by overcoming S1PR1-mediated immune egress [18]. Following activation, T cells go through several rounds of cell division. After activation and clonal expansion of lymphocytes, S1PR1 are upregulated again and CCR7 expression is downregulated, following stimulation by S1P secreted by lymphatic vascular cells expressing lymphatic vessel endothelium receptor-1. This, in turn, leads to the egress of lymphocytes in order for them to home in on target organs for immune regulatory functions [7]. The expression of CD69 on effector CD8 T cells that counteract the S1PR1 effect is necessary for T cell to stay in local tissues to perform adaptive immune response [18]. 

**Figure 2:**
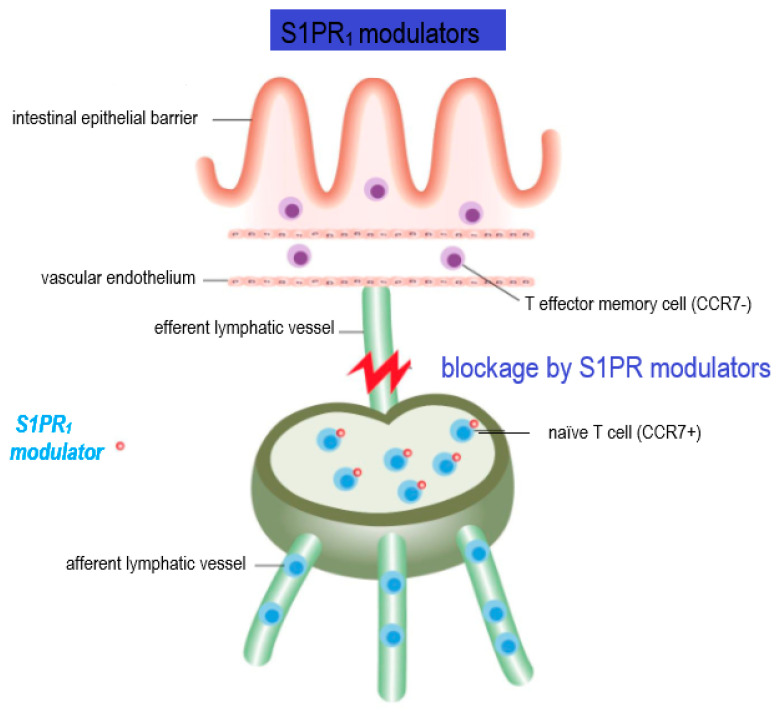
Mechanism of action of S1PR modulators. The figure illustrates the blockage of T cell egress by S1PR modulators, which is the core of their therapeutic effect in MS. CCR7, a chemokine receptor expressed in naïve and central memory T cells, retains lymphocytes within lymph nodes by overcoming S1P1-mediated immune egress. Following activation and clonal expansion, T cells switch to a state favoring egress over retention by downregulating CCR7. Importantly, effector memory cells do not express CCR7 receptors and have the ability to overcome S1P regulation.

As mentioned above, not all T cell subpopulations’ trafficking is affected by S1P signaling within the lymph nodes. S1P−S1PR1 action can overcome the retention signals mediated by the chemokine receptor CCR7, which is expressed by naïve T cells, central memory T cells, and B cells. In contrast, terminally differentiated effector T cells and effector memory T cells do not express CCR7 receptors, therefore they have the capacity to egress from lymph nodes independent of S1P−S1PR1 signaling [7,21,22]. This specific property of T effector and T effector memory cells to overcome S1P regulation has crucial implications in clinical practice, where the administration of S1P receptor modulators, such as fingolimod does not inhibit these cells from recirculating to peripheral tissue and performing important immunosurveillance functions [7] (Figure 2).

Similarly, S1P gradient-dependent regulation is used for Β cell sequestration in secondary lymphoid organs. The expression of different S1P receptors in various human B cell populations is responsible for the regulation of their circulation. S1P signaling via S1PR1 drives the transferring of newly generated immature B cells from the bone marrow to the blood [18]. S1PR1 activation overcomes the recruiting signal of CXCL13 and also promotes B cell egress from the follicles and localization to the marginal zone. In humans, the S1PR1-dependent signaling for the circulation of B cells is carried out by interaction with β-arrestin 2, LPS-responsive beige-like anchor protein, dedicator of cytokinesis 8, and Wiskott−Aldrich syndrome protein. Upregulation of S1PR2, on the other hand, enhances the centering of activated B cells in the follicle and the confinement of germinal center B cells in the germinal center through interaction with Ga13 [7]. Studies have also shown that S1P signaling mediates T helper (TH) cell development and differentiation, including TH1/TH17 development and maintaining TH1/ T regulatory cell (Treg) balance [18,23].

### 2.2. Regulation of Innate Immune Cell Trafficking

S1P signaling controls trafficking of the other immune cells, including dendritic cells [15,24], natural killer cells [25,26] and hematopoietic stem cells [11,27,28]. S1PR1 is responsible for neutrophil migration [21]. In addition, it mediates neutrophil recruitment, as was demonstrated through a rat model of hyperalgesia [29], and a model of *Candida albicans*-induced vasculitis, where the administration of an SPR1 agonist, ONO-W061 suppressed neutrophil recruitment [30]. The recruitment of macrophages, eosinophils and mast cells is also regulated by S1PR1 activation. In macrophages, S1PR1 also evokes the anti-inflammatory response and serves the signaling required for cell apoptosis [16]. A study in myeloid specific S1PR1-deficient mice observed prolonged survival of macrophages and the inhibition of apoptosis, both in vitro and in vivo [31]. Moreover, S1PR1 expression is required for control of trafficking of monocytes and dendritic cells, as well as for the inhibition of IFN-a secretion, by dendritic cells. [16]. S1PR2 is expressed on the surface of macrophages, monocytes and granulocytes. Studies of infected S1PR2 macrophages with *Cryptococcus neoformans* [32] and *Escherichia coli* [33] have revealed an important role for S1PR2 in increasing antibody-mediated phagocytosis of fungi and inhibiting phagocytosis of bacteria in alveolar macrophages. S1PR2 is also important for the triggering of mast cells and enhancement of degranulation, during viral infection. An interesting, recent finding about S1PR2 indicates that its activation by intracellular S1P leads to phosphorylation of ezrin-radixin-moesin (ERM) proteins, which play an important role in phagocytic cell function [16,34]. 

In innate cells, S1PR3 is present on multiple cell subtypes. It promotes dendritic cell maturation. It drives macrophage chemotaxis and killing, as shown by studies in vitro and in vivo where S1PR3-mediated macrophage action caused atherosclerosis via altering smooth muscle cell behavior [35]. S1PR3 also mediates neutrophil and eosinophil recruitment in models of asthma [7]. It also drives leukocyte rolling on endothelial cells [16]. S1PR4 is expressed on most immune cells, and current research suggests a role in plasmacytoid dendritic cell differentiation and activation, as evidenced by studies in S1PR4 knockout mice [36]. It regulates neutrophil migration from blood to tissue [21,28], and has important roles in macrophages as well. Interestingly, it was found that S1PR4 is the only receptor that displays a significant difference in its expression between M1 and M2 states of activated macrophages, suggesting an important role for this receptor in multiple macrophage actions as migration and cytokine release [37]. Last, the S1PR4-mediated regulation of cytokine production by myeloid cells appears to be the central function of this receptor, compared to its less active role in immune cell migration [28]. The co-expression of CXCR4 and S1PR5 is a prerequisite for mature natural killer cells to leave the lymph nodes and bone marrow [16,26]. S1PR5 is also required for the recruitment of NK cells to sites of inflammation [7,16]. Patrolling monocytes, similar to NK cells, require S1PR5 to egress from the bone marrow. However, unlike T cells, they do not require S1P gradient for their trafficking, suggesting that S1PR5-mediated trafficking is controlled by a different mechanism [16,38]. Recent findings have suggested that S1PR1-myeloid cell signaling is considered to impact the initiation and progress of CNS autoimmunity [39]. Studies have focused on unravelling their role in CD14 and CD16 myeloid cells [40]. (Table 1)

## 3. S1P Signaling in the Intestine

The gastrointestinal (GI) tract is the largest epithelial barrier that protects the human body from the external environment. Preservation of an intact intestinal mucosal barrier is important for GI functions including digestion of food, absorption of nutrients, expulsion of waste, and protection against hostile bacteria [41]. The proliferation and migration of intestinal epithelial cells (IECs) must be carefully balanced and regulated [42]. This way, the body can preserve the integrity of the intestinal mucosal barrier and manage to maintain the protection of the host from the environment but also the communication between host and its environment. These functions are regulated by a variety of signaling molecules [43]. The intestinal immune system must preserve its immunological homeostasis. The disturbance of the intestinal immune function causes the outbreak of allergic, inflammatory, and infectious diseases [44,45]. S1P is a biolipid mediator that controls various cell functions, as described in detail above. In the gastrointestinal system, S1P has important roles in maintaining intestinal epithelial cell barrier structural and functional integrity, regulating IEC proliferation, migration and apoptosis. It also mediates immunological actions like immunoglobulin A production and T cell trafficking [41,44,45]. In general, S1P is a byproduct of the catalyzation of sphingomyelin and is produced mainly by platelets, erythrocytes, and endothelial cells in mammals. However, in the intestinal tissue the production of S1P is mainly due to epithelial cell activity.

Interestingly, it has been suggested that the levels of dietary sphingomyelin intake and the activity of the enzymes sphingomyelinase and sphingosine kinase may influence the incidence and severity of intestinal inflammatory processes [44,46,47,48]. Furthermore, cholesterol ingested by food antagonizes the intestinal absorption of sphingolipids. Thus, it is implied that modern Western diets, rich in cholesterol, may promote S1P-mediated intestinal immunity dysfunction and the subsequent genesis of inflammatory disorders, through its impact in reducing the availability of S1P precursors [45,49]. Recent studies have brought up evidence supporting the role of S1P signaling in the activation of two critical transcription factors, nuclear factor kappa B (NF-κB) and signal transducer and activator of transcription 3 (STAT3). These transcription factors are known to regulate key inflammatory signaling pathways that promote colon inflammation and carcinogenesis in the intestinal as well as in other human systems [50]. 

### Implication in Intestinal Barrier Homeostasis

S1P is found abundantly in the human intestine. All five S1PRs are expressed in intestinal epithelial cells, as indicated by the findings from the Human Protein Atlas, but the expression level of each one varies, with the highest being that of S1PR2 [51]. The role of S1P in intestinal epithelial barrier integrity has been investigated, but still not all its functions have been revealed. Greenspon et.al have demonstrated that, following S1P administration in differentiated rat IECs, intestinal barrier function was enhanced. Treatment with S1P led to an increase in expression of an adherens junction protein, E-cadherin, and also in more efficient redistribution of this protein on the barrier. Increased abundance of S1P, resulting from upregulated expression of SphK1 in IECs, was also related to augmented levels of other barrier proteins including claudin-1 and occludin. These effects were attributed to activation of S1PR1 [52]. Chen et al. have shown that S1P also upregulates the expression of c-Myc, cyclin D1, E-cadherin and zona occluden-1 (ZO-1) via activation of S1PR2 [43]. These effects result in maintaining the integrity of the intestinal epithelial cell barrier. The above authors later conducted further research experiments, using an S1PR2-knockout mice model, with the administration of Dextran sulfate sodium (DSS) to induce colitis. The pivotal role of S1P-mediated S1PR2 activation in regulating intestinal barrier structural integrity and function was reaffirmed. It was demonstrated that the S1P/S1PR2 axis in IECs prevented intestinal barrier damage by mediating (a) CD4+T-cell activation via the ERK pathway and (b) MHC-II expression. In addition, they found that IFN-γ, secreted by CD4+T cells, augmented DSS-induced damage of the intestinal barrier function. This was done through downregulation of ZO-1 by IFN-γ. On the other hand, S1P was capable of restricting DSS/IFN-γ-induced damage of intestinal mucosa permeability by increasing ZO-1 [53]. Additionally, S1P has been found to promote intestinal epithelial cell proliferation and migration, in a dose-dependent manner. This is achieved through S1P-mediated activation of ERK1/2 via S1PR2 [43]. Furthermore, S1P-mediated activation of S1PR2 leads to increased expression of down-regulated in adenoma (DRA), the major Cl-/HCO3-exchanger that regulates NaCl absorption in the intestine of mammals [54]. MEK/ERK activity controls JNK activation via regulating MAPK phosphatase (MKP-1), which prevents cell apoptosis. S1P enhances the ERK1/2 and Akt signaling pathways thus preventing intestinal epithelial cells’ apoptosis [55,56] (Table 1).

## 4. S1P Signaling in the CNS

S1P is widely present in the CNS, where it is released by cerebral sphingosines and acts on S1P receptors. All S1PRs except S1PR4 are expressed in the CNS by neuronal and glial cell populations and also in the cerebral vascular system, to varying degrees. Their activation by S1P mediates numerous physiologic processes involved in neuronal plasticity including myelination, neurogenesis and neuroprotection [57]. Evidence from animal models and studies in vitro suggest that S1P regulates multiple physiologic functions in the CNS and that the expression of S1PRs is dynamic, influenced by temporal and spatial factors and stimuli of the cell environment [4,58,59].

In neurons, S1P signaling has an active role in neural progenitor migration, synaptic activity (=neurotransmission), differentiation, process extension, calcium signaling and survival [60]. S1P stimulates neurogenesis more effectively than fibroblast growth factor (FGF) [61]. S1P binding to S1PR1, S1PR2 and S1PR5 participates in the regulation of growth cone formation, neurite extension and retraction. In vitro experiments of stimulation of neurons with nerve growth factor (NGF) showed the resulting enhancement of neurite extension which is mediated by S1PR1. Opposingly, the activation of S1PR2 and S1PR5 inhibits neurite extension [62]. Several researchers have addressed the role of S1P-mediated circuits in neural progenitor stem cells (NPSCs). The effect of S1P on NPSCs is mediated by increased laminin expression and extracellular matrix (ECM) interactions with progenitor integrins [63]. NPSCs that migrate out of the embryoid body stem cells (ESCs) are considered to upregulate S1PR1 [64]. However, there are important variations between studies concerning the description of S1PR expression in NPSCs [61]. S1PR1 also affects plasma membrane excitability and neurotransmitter release, thus promoting synaptic transmission, as demonstrated in studies with rat hippocampus models [65,66]. Control of neural activity has been attributed to S1PR2 signaling [67], as models of S1PR2-knockout mice have resulted in excess neural excitability and the occurrence of seizures at 3–7 weeks of age [60,68]. The regulation of synaptic activity, neurogenesis and cell survival is such an important factor that these receptors have attracted interest as potential drug targets in memory disorders and neurodegenerative diseases. 

Oligodendrocytes and oligodendrocyte precursor cells (OPCs) widely express S1PRs, and the level of each S1PR’s expression depends on the cells’ stage of development and on the myelinating state [58,69,70]. In mature oligodendrocytes, S1PR5 expression is highest and is thought to promote cell survival via the S1PR5-mediated Akt signaling pathway and inhibit OPC migration via the S1PR5-mediated Rho GTPase/Rho kinase pathway [71], whereas it is assumed that early stages of differentiation are mainly mediated by S1PR1 signaling [59]. Indeed, various authors suggest that higher expression level and activation of S1PR1 induce the differentiation of oligodendrocytes along with the survival of oligodendrocyte progenitors [61,72]. Regarding the role of S1P in oligodendrocyte morphology, there is an interesting dual action. Via the modulation of S1PR5, it enhances process retraction [73], while via S1PR1, it promotes process extension, indicating reciprocal effects on cytoskeletal elements. S1PR5 is also considered to have a pivotal role in myelination, although data from S1PR5-null mice showed that myelination was normally developed despite the lack of S1PR5 expression [73]. On the other hand, the targeted deletion of S1PR1 in oligodendrocyte lineage cells led to abnormal formation of myelin and increased susceptibility to cuprizone-induced demyelination [74]. This finding suggests an important role for S1PR1 in normal myelination of the brain. S1P actions on oligodendrocytes and OPCs are also influenced by the action of neurotrophic factors and the lysophosphatidic acid receptors expressed on these cells, such as neurotrophin-3 and platelet-derived growth factor. It seems very probable that the process of myelination is coordinated with the contribution of all the above cellular structures [60,75]. Altogether, interactions between S1P and S1PRs in oligodendrocytes and OPCs control cell survival, migration and differentiation, maintenance of cell morphology, myelination and remyelination. 

S1P signaling mediates various functions in astrocytes, including differentiation [61,76], proliferation, migration, gap junction communication, growth factor production and astrogliosis [59,60]. Astrocytes mainly express S1PR1 and S1PR3, with scarce expression of S1PR2. S1PR5 expression by astrocytes was detected during in vitro studies, when exposed to growth factors [77]. Astrocyte proliferation is stimulated by S1P via activation of the extracellular-signal-regulated kinase (ERK) pathway [78]. Observations of activated astrocytes in response to pathogens suggest that they overexpress S1PR1 and S1PR3, pointing to a role of these receptors in astrogliosis [79]. Studies in S1PR-knockout mice also suggest that astrogliosis requires the activation of S1PR1 and S1PR3 [80]. Some research groups support that S1PR3 is overexpressed in astrocytes under proinflammatory conditions [61,76]. Interestingly, S1P antagonism by fingolimod reduces the production of the inflammatory chemokines CXCL5/LIX, C-X-C motif chemokine 10 (CXCL10) and monocyte chemoattractant protein-1 (MCP-1) in astrocytes and microglial cultures [81]. Therefore, S1P signaling in astrocytes seems to mediate both proinflammatory and anti-inflammatory effects. 

S1PR1, S1PR2 and S1PR3 are also expressed by microglia. Microglia exist in a nonactivated state and switch to an activated state when stimulated by proinflammatory cytokines. The expression of S1PRs in microglia cells depends on their activation state [82]. Extracellular S1P is a powerful chemoattractant for microglial cells in the brain [61]. S1P levels have been shown to increase at sites of brain damage, where microglia and neural progenitors accumulate. S1PR1 and S1PR3 are downregulated in activated microglia, and S1PR2 is upregulated. In models of experimental autoimmune encephalomyelitis (EAE), S1PR1 deletion was correlated with reduced activation of microglia cells [81]. (Table 1)

### Implication in BBB Homeostasis 

We have already discussed the effects of S1P signaling in astrocytes, whose projections surround the endothelial cells of the blood−brain barrier (BBB). Apart from that, the regulation of endothelial adherens junctions by S1P signaling also plays a critical role in the maintenance of the vascular barrier integrity [6,11]. A compromised endothelial barrier results in increased vascular permeability, one of the core features of inflammation, tumor metastasis and atherosclerosis. S1P signaling strengthens the adherens junctions between endothelial cells to limit exaggerated inflammation. On the other hand, proinflammatory mediators including histamine and leukotrienes enhance the inflammatory response by loosening of the adherens junctions to allow the excretion of antibodies and complement, and the attraction of leukocytes and lymphocytes to the site of inflammation [11]. Plasma S1P-knockout mice display increased morbidity due to increased vascular leak and anaphylaxis following the administration of platelet-activating factor or histamine [83]. Likewise, pharmacologic blockage of S1PR1 or ApoM deficiency leads to compromised vascular integrity and excess inflammation [84]. Brain endothelial SP1 signaling also supports the BBB integrity by arranging the localization of tight junction proteins [85]. Maintenance of vascular integrity requires proper rearrangements of the cytoskeleton as well as the assembly of adherens junctions in endothelial cells. S1P promotes the structural integrity of the actin cytoskeleton, the accumulation of VE-cadherin and α-, β- and γ-catenin at the sites of adjacent cell contact, and adherens junction assembly [86]. The activation of small G proteins Rac and Rho downstream of the S1PR1 and S1PR3 signaling pathways are involved in the processes [86,87]. S1P−S1PR1 signaling is considered to have a pivotal function in regulating the inflammatory status of vascular endothelial cells. Giovani et al. have shown that endothelial S1PR1 abundance was enhanced in regions of vascular inflammation. Additionally, proinflammatory adhesion proteins such as VCAM-1 and ICAM-1 were upregulated in the descending aorta of mice with endothelial cell-specific deletion of S1PR1 and suppressed in mice with endothelial cell-specific overexpression of S1PR1 [12]. These observations indicate that proper S1PR1 localization and signaling are important to maintain vascular homeostasis, and that the impairment of S1PR1 signaling because of receptor internalization predisposes endothelial cells to an inflammatory phenotype [11].

## 5. Implication for the Role of S1P in Multiple Sclerosis

The ubiquitous presence of S1P and its receptors in the CNS implies that the S1P−S1PR signaling system may be targeted for therapeutic purposes in neurological disorders like MS [88]. MS is a T cell-mediated autoimmune disease of the CNS and its etiology is believed to constitute an interaction between genetic predisposition and environmental factors. MS is classified as follows, according to the clinical course of the disease: relapsing–remitting MS, primary progressive MS, and secondary progressive MS [89].

The activation and clonal expansion of CNS-directed, autoreactive CD8+ T cells, differentiated CD4+ TH1 and TH17 cells, B cells and innate immune cells in the periphery are considered to be the first step in MS pathogenesis [90]. Following this, T cells cross the BBB, with the interference of integrins and, upon entrance, they are reactivated by epitopes on myelin and induce inflammation with the release of cytokines such as tumor necrosis factor alpha (TNFα) and IFNγ. These events lead to increased BBB permeability, demyelination and axonal degeneration [89,91]. After their activation by TNFα and IFNγ, CNS resident microglia and infiltrating macrophages are transferred to the site of inflammation, also contributing to destruction of myelin and prolonging neurodegeneration [92]. The B lymphocytes’ role in the pathogenesis of the disease has attracted vivid interest and thorough research in the last years, and therapeutic agents targeting these cells have shown positive results in limiting disease progress [93]. B cells contribute to MS pathophysiology through multiple mechanisms such as antigen presentation to T cells, transport of antigens from tissues to secondary lymphoid organs and the release of proinflammatory or anti-inflammatory cytokines. Pathogenetic auto-antibodies have been identified in a subgroup of MS patients [94]. 

Following the acute inflammatory attack, the patient exhibits functional recovery that is attributed to the remyelination of the axons and brain plasticity. However, axonal damage, which is secondary to demyelination and neurotoxic factors released from activated microglia, is not reversible. Accumulating axonal damage leads to neuronal degeneration and brain atrophy, which is the main contributor of progressive irreversible neurological disability [57]. Another residue of the acute inflammatory event is the subsequent chronic inflammation which is restricted within the CNS. This is due to the persistence of activated immune cells in the CNS, despite the absence of infiltrating lymphocytes from the periphery. This chronic inflammatory process affects the whole brain parenchyma, even at sites far from the underlying focal demyelinating lesions. Diffuse chronic CNS inflammation is considered more prevalent in patients with progressive forms of MS [57,95]. Inflammation in MS is thus differentiated both in the space axis (peripheral vs. local CNS inflammation) and in the time axis (acute vs. chronic inflammation). Both inflammatory and neurodegenerative components of the disease are thought to be present from the early stages, and proceed in parallel, but are responsible for different aspects of the clinical phenotype. 

## 6. The Role of the Gut–Brain Axis in MS Course and Progression

The complex relationship between increased intestinal permeability, gut microbial dysbiosis and autoimmunity has significant implications in the pathogenesis and development of immune-mediated diseases including MS and other entities, such as inflammatory bowel disease (IBD). The implication of S1P signaling in the intestinal barrier and BBB homeostasis is important because dysregulation of the barriers is an integral part of many disorders of the gut and the brain. These organs maintain a two-way communication system, which has been described as the gut–brain axis and it is regulated at neural, endocrine and immune levels [96]. The intestinal barrier, with its physical, biochemical and immunological properties, prevents direct exposure of the host immune system to the microbiota, thus limiting the initiation of unwanted immune responses [97]. Gut microbiota, part of the intestinal ecosystem, has gained attention because of its role in regulating both local and systemic immune responses [98]. Interestingly, it has been shown that some bacteria secrete metabolites that could modulate the S1P axis. A recent study has shown that LPS along with palmitate, a major saturated fatty acid, can stimulate proinflammatory gene expression by increasing the production of both ceramide and S1P [99]. The critical role of the S1P axis at the host−pathogen interactions has been further highlighted by recent data. In particular, *Burkholderia*-encoded S1PLs are important for *Burkholderia pseudomallei* and *Burkholderia thailandensis* virulence and intracellular survival [100]. Alterations in the gut microbiota, defined as alterations in the relative composition of the microbial community, are a common underlying condition in MS, but whether they precede the disease or it is the disease that affects the function and composition of microbiota is a matter of debate [101]. A recent systematic review of studies with respect to the composition of the intestinal microbiota in patients with MS concluded that there is no statistically significant difference in the diversity of the intestinal microbiome in MS patients compared to normal controls. However, taxonomic differences were observed, with common patterns of intestinal dysbiosis in patients with MS [102]. Key findings from taxa-level relative abundances include a higher relative abundance of *Akkermansia* and *Methanobrevibacter* and a lower relative abundance of *Prevotella, Bacteroides* and *Faecalibacterium prausnitzii* for MS cases compared to controls. *Akkermansia muciniphila* has previously been reported to have proinflammatory functions in vitro, by promoting Th1 lymphocyte differentiation [103]. Another study indicates that *Akkermansia muciniphila* can exacerbate intestinal inflammation in *Salmonella Typhimurium*-infected mice, probably through its ability to disturb mucus layer homeostasis [104]. On the other hand, *Prevotella histicola* has been shown to attenuate inflammation and suppress disease activity in an animal model of MS by inducing CD4+ FoxP3+ regulatory T cells [105]. In addition, oral administration of a single purified polysaccharide antigen derived from *Bacteroides fragilis* has been shown to protect mice from demyelination in an IL-10-dependent mechanism [100]. In spite of the aforementioned important observations, a causal relationship between microbial pattern alterations and MS onset or outcomes has not yet been established. As far as the intestinal barrier in concerned, clinical and experimental evidence have shown alterations in intestinal permeability in patients with MS. For example, a pilot study that applied the lactulose/mannitol permeability test to evaluate intestinal permeability in 22 patients with multiple sclerosis compared with controls, concluded that the proportion of participants with increased permeability was significantly higher in patients than in controls [106]. Other findings from an animal model of MS suggest that the disturbance of intestinal homeostasis is an early and immune-mediated event in EAE [107]. Furthermore, Secher et al. observed a correlation between the degree of intestinal permeability disruption and EAE severity [108]. Increased intestinal permeability (“a leaky gut”) is associated with low-grade chronic microbial translocation and elevated lipopolysaccharide (LPS) levels in systemic circulation, a condition known as endotoxemia that is involved in the development of MS as it leads to chronic systemic inflammation [102]. Circulating inflammatory factors compromise the integrity of the BBB and ultimately reach the CNS where they can activate microglia and astrocytes, further promoting the inflammatory environment in the context of autoimmunity [109]. Pharmacologic interventions targeting the restoration of the altered intestinal barrier may limit the exposure of immune cells to microbial derivatives and thus reduce the associated proinflammatory cascade [110].

## 7. S1PR Modulators in MS Therapy

### 7.1. Immunomodulatory Effects

The first S1PR modulator that was approved as an immunomodulatory therapeutic agent for human use is fingolimod (FTY720), for relapsing−remitting MS [111]. It was first created as a potent immunosuppressor, by chemical modification of myriocin, a metabolite of the fungus *Isaria sinclairii*, that was used as a remedy in Eastern traditional medicine [112]. Unlike other immunomodulatory agents, FTY720 does not inhibit proliferation and activation of T and B cells, instead it acts on prohibiting the circulation of lymphocytes [113]. FTY720 has a significant degree of structural similarity with sphingosine and exerts its actions via binding to all S1P receptors except S1PR2. In vivo, FTY720 becomes phosphorylated to FTY720-P by sphingosine kinase 2, that is its active form [11,114].

The basis for FTY720′s immunosuppressive action is the functional antagonism of S1PR1. FTY720-P binds to S1PR1 inducing desensitization of the last, followed by sustained internalization, WWP2 (ubiquitin E3 ligase)-dependent polyubiquitinylation and consequent degradation and reduced expression of the receptor. By limiting S1PR1 expression, FTY720 inhibits the lymphocyte egress from lymphoid organs, leading to lymphopenia, which is the core of its therapeutic effect in MS [11]. Aside from this prominent effect, other peripheral immunomodulatory actions of fingolimod that may also contribute to its efficacy are investigated. Reduced release of proinflammatory cytokines from dendritic cells, with implications on T cell activation from these antigen-presenting cells, was demonstrated in patients with MS treated with fingolimod, on a slower timescale compared to the direct effect of lymphopenia [115]. Another study examined the effects of fingolimod on modulation of T cell phenotypes in vivo. Interestingly, the treatment inhibits the Th1 phenotype and the expression of proinflammatory cytokines such as IL-17 and IFNγ on CD4+T cells, while increasing the production of anti-inflammatory cytokines such as TGFβ and IL-10 [116]. An anti-inflammatory cytokine profile of B cells along with an increased proportion of regulatory B cell subsets were also observed in fingolimod-treated patients [117]. The aforementioned data suggest a peripheral immunosuppressive action of fingolimod in MS treatment. Interestingly, fingolimod further acts on circulating and CNS-resident myeloid cells by suppressing their inflammatory activity (release of proinflammatory cytokines such as TNFα, IL-1β or IL-6) [118].

Second-generation S1PR modulators which have been approved for the treatment of relapsing forms of MS include siponimod, ozanimod and ponesimod. These modulators differ from fingolimod in various ways, including their selectivity for S1PR subtypes, smaller half-lives, an improved safety profile and no requirement for in vivo phosphorylation [119]. Siponimod and ozanimod are specific for S1PR1 and S1PR5 whereas ponesimod has high affinity for S1PR1 [120]. Ceralifimod and amiselimod which are selective S1PR1 and S1PR5 modulators, have been successfully tried in phase II clinical trials but their further clinical development was discontinued [121]. The cardiac chronotropic effects of siponimod and ozanimod can be easily managed with dose-titration strategies, generally avoiding the need for first dose observation (FDO) [119]. Improved bioavailability along with higher specificity of second-generation modulators may also maximize their direct CNS effects and clinical benefit, including in progressive forms of MS [119].

### 7.2. Potential Neuroprotective Effects

Besides blocking T cell egress, the neuroprotective and central anti-inflammatory effects of S1PR modulators are under investigation. Of note, fingolimod can easily cross the BBB due to its lipophilic nature and exert effects on cells within the CNS [122]. Studies in animal models of MS have shown that fingolimod has the potential to affect proliferation and differentiation of OPCs, thus facilitating remyelination [123]. Interestingly, in vivo, the combined treatment of fingolimod and neural stem cells (NCSs), enhances remyelination and promotes CNS repair processes in EAE models via driving NCS differentiation into OLGs [124]. It seems that these neuroprotective effects are demonstrated independently of fingolimod’s immunomodulatory capacity [125]. Another important observation is that the effectiveness of fingolimod in EAE models requires the expression of S1PR1 in astrocytes [126]. Additionally, administration of fingolimod in astrocyte cultures from EAE models has led to a reduction of chronic CNS inflammation, as a result of suppression of proinflammatory cytokines and neurotoxic factors, such as IL-1, iNOS and nitrotyrosine [127]. This modulation of astrocyte-derived factors by fingolimod also has an impact on microglia polarization, further regulating the inflammatory milieu inside the CNS [128]. A significant amelioration of microgliosis and astrogliosis was observed following the administration of siponimod in EAE models [129]. Moreover, siponimod therapy in a preclinical model of subpial cortical injury reduced the production of Th 17 cytokines by T cells, leading to a reduction in demyelination and the accumulation of microglia [130]. Another study concluded that siponimod positively affects cortical network functionality in a mouse model of focal EAE [131]. Dysregulation of synaptic transmission has been highlighted in the pathophysiology of the disease, leading to degenerative neuronal damage. Administration of fingolimod and siponimod in animal models of MS rescued defective synaptic transmission and prevented synaptic degeneration [129,132]. Siponimod interference with astrocyte-induced degeneration has also been investigated. In vitro models of astrocytes generated from human fibroblasts and on spinal neurons exposed to astrocyte-conditioned media have described that, siponimod targeting of S1P receptors resulted in the inhibition of NFκB translocation and enhancement of nuclear translocation of Nrf2. These events led not only to the direct restriction of neuroinflammation but also to the protection of neurons from astrocyte-induced degeneration [133]. Moreover, ozanimod has also been shown to exert neuroprotective actions, via S1PR1 binding, in EAE brain specimens and microglial cell cultures, by promoting amelioration of EAE-driven striatal glutamatergic synapse alterations [134]. 

These findings indicate that S1PR modulators may also target the neurodegenerative component of the disease along with the inflammatory one. Table 2 summarizes the main animal models in MS studying the effects of S1P modulators.

### 7.3. Additional Effects under Investigation

Additional mechanisms of action of these drugs include bactericidal properties that may contribute to alterations in the composition of gut microbiota. This is an important observation, since it has been proposed from previous studies that epsilon toxin, from *C. perfringens*, is a potential causative agent for newly forming MS lesions [139,140,141]. Both sphingosine and fingolimod proved to be potent *C. perfringens* inhibitors in vitro, resulting in reduction of the associated endotoxin production [142]. It is also suggested from other studies that fingolimod may impact intestinal immunity through the regulation of B cell trafficking and IgA plasmablast maturation [143]. A recent study in nonobese diabetic (NOD) mice showed that early-life fingolimod treatment has beneficial effects on the intestinal homeostasis such as enhancement of intestinal barrier integrity, attenuation of microbial dysbiosis and improvement of defective intestinal immune function. It also proved that fingolimod administration improves pancreatic islet immune tolerance in NOD mice [144]. Further study of S1PR modulators-derived potential effects in the intestinal barrier and gut immune system could support the understanding of the MS disease course. Apart from MS, S1PR modulators represent a promising therapeutic strategy for other immune-mediated diseases, including IBD [136,145], mainly via amelioration of lymphocyte trafficking across the intestine (Table 3). Mocravimod, ozanimod, etrasimod and amiselimod have been all tested successfully in colitic animal models [146]. Ozanimod has been shown to reduce inflammation and disease parameters in three models of autoimmune disease, including colitis [136]. Τhe results of open-label extension (OLE) of the TOUCHSTONE clinical trial in patients with moderate to severe ulcerative colitis demonstrated that ozanimod has significant benefits and long-term efficacy based on clinical, endoscopic and biomarker measures for up to 4 years of treatment [147]. Endoscopic, histological and clinical improvement was also observed within 12 weeks after initiation of ozanimod in STEPSTONE clinical trial in patients with moderately to severely active Crohn’s disease [148]. These insights can direct the therapeutic application of these drugs in other human disease areas.

## 8. Conclusions

S1P signaling mediates a wide range of physiological and pathophysiological cell functions, through the activation of sphingosine 1-phosphate receptors 1–5. S1PRs are ubiquitously expressed by CNS, immune, intestinal, cardiac and vascular cells. S1P/S1PR-mediated biological pathways are pivotal for cell proliferation, angiogenesis, neurogenesis and, importantly, for the egress of lymphocytes from secondary lymphoid organs. Its multifactorial role in inflammation and immunity, both innate and adaptive, implies an involvement of S1P signaling in the pathogenesis of autoimmune disorders, like MS. The wide expression of S1P and its receptors in the CNS further support the notion that the S1P−S1PR signaling system is involved in the pathogenesis of MS. Therapeutic targeting of S1PRs in MS exerts multiple effects through the modulation of the immune, the intestinal and the central nervous system. In addition to reducing the invasion of autoreactive lymphocytes in the CNS, fingolimod, the first S1PR modulator that was approved for the treatment of MS, easily crosses the BBB and has direct neuroprotective actions including the preservation of BBB integrity and decreasing the secretion of proinflammatory cytokines by astrocytes and microglia. Each of the key resident CNS cells involved in MS pathogenesis can be regulated by S1PR modulators. Moreover, the inherent processes of MS pathology, such as astrogliosis and demyelination, are mediated by S1P signaling in the CNS [155]. As highlighted above, S1P signaling also regulates intestinal barrier and BBB integrity. A dysfunctional intestinal barrier along with dysbiotic changes in the gut microbiota have been described in MS patients and thus, modulation of the microbiota−gut–brain axis by these agents may contribute to their overall therapeutic efficacy. Stabilization of the intestinal barrier along with restoration of a dysbiotic gut microbiota could potentially limit endotoxemia and subsequently, chronic systemic inflammation. Further studies are needed to investigate the value of such actions of S1PR modulators and whether they actually play a meaningful role in clinical response. 

With respect to future perspectives, a better understanding of the direct neuroprotective and regenerative effects of S1PR modulators would provide some insight for the administration of these agents to patients with progressive forms of MS. In this case, the neurodegenerative component of the disease would be targeted along with the inflammatory one. Along with further experimental studies on the above issue that are expected to run in the following years, real-world evidence from the clinical experience on the administration of S1PR modulators will accumulate as well. Patient data derived from cohorts of people currently being treated with S1PR modulators might shed light on the neuroprotective effects of these agents, when examined in retrospective. 

Moreover, the complex relationship between the gut–brain axis and MS should be delineated through additional research. Future studies should focus on addressing the effects of S1P modulators on gut microbiota in EAE animal models. As mentioned above, preliminary data supports that some bacteria secrete metabolites that could modulate the S1P axis. Additional research must delineate whether certain gut microbiota populations have S1PR-modulating properties, through preclinical animal or cell models. 

Lastly, the multiple and complex effects of fingolimod in various cell types suggests that S1PR modulators may, hopefully, be effective in treating CNS diseases other than MS as well as systemic autoimmune disorders. 

## Figures and Tables

**Table 1:** S1P receptor expression in cells of immune, gastrointestinal and nervous system and associated cell functions.

Receptor	Associated Cell Types	Functions (Described up to Date)
S1PR1	Immune system: T cells, B cells Macrophages Neutrophils DCs Monocytes Eosinophils Mast cells NK cells	Egress from lymph nodes, exit of mature T cells from thymus, migration of natural killer T cells from secondary lymphoid organs to circulation, transfer of immature B cells from bone marrow to circulation Macrophage recruitment Neutrophil migration, recruitment Trafficking of DCs Trafficking of monocytes Eosinophil recruitment Mast cell recruitment
	GI: IECs	Upregulation of intestinal barrier proteins (claudin1, occludin)
	CNS: Astrocytes Oligodendrocytes Neurons Microglia	Activation, differentiation, proliferation of astrocytes, astrogliosis Differentiation of oligodendrocytes, process extension, survival of oligodendrocyte progenitors, myelination Growth cone formation, enhancement of neurite extension, synaptic transmission
S1PR2	Immune system: Macrophages Monocytes Mast cells Eosinophils	Enhance antibody-mediated phagocytosis, inhibit phagocytosis of bacteria and fungi Degranulation of mast cells
	GI: IECs	Upregulation of c-Myc, cyclin D1, E-cadherin and Zona occludin 1, proliferation of IECs, absorption of NaCl, prevention of IECs apoptosis
	CNS: Neurons Microglia	Growth cone formation, inhibition of neurite extension, control of neural excitability
S1PR3	Immune system: Macrophages Monocytes Neutrophils DCs Eosinophils Mast cells	Leucocyte rolling on endothelial cells Macrophage’s chemotaxis and killing Neutrophil recruitment DC maturation Eosinophil recruitment
	GI: IECs	
	CNS: Astrocytes Microglia	Activation, differentiation, proliferation of astrocytes, astrogliosis
S1PR4	Immune system: Macrophages Monocytes Neutrophils DCs Eosinophils Mast cells	Macrophage migration and cytokine release Neutrophil migration Plasmacytoid DC activation and differentiation
	GI: IECs	
	CNS:	
S1PR5	Immune system: Patrolling monocytes NK cells	Egress of mature NK cells from lymph nodes and bone marrow
	GI: IECs	
	CNS: Mature oligodendrocytes Neurons	Cell survival, process retraction, inhibition of OPC migration, myelination Growth cone formation, inhibition of neurite extension

**Table 2:** S1P modulators in EAE and cuprizone models of demyelination.

Authors	Drug	MS Model	Main Findings
Choi et al., 2011 [126]	Fingolimod	EAE	The effectiveness of fingolimod is mediated by modulation of S1PR1 in astrocytes
Rossi et al., 2012 [132]	Fingolimod	EAE	Ameliorates pre- and postsynaptic glutamatergic transmission and restores clinical signs of disease
Colombo et al., 2014 [127]	Fingolimod	EAE	Fingolimod suppresses astrocytic activation (S1P, IL17, and IL1 induced NFκB translocation and NO production) and prevents astrocyte-induced neuronal death
Di Dario et al., 2015 [118]	Fingolimod	EAE	Suppression of release of proinflammatory cytokines (TNF-α, IL1β, IL6) leading to inhibition of myeloid cell activation (both in periphery and CNS)
Zhang et al., 2015 [123]	Fingolimod	EAE	Promotion of OPC proliferation and differentiation, decrease in disease severity
Zhang et al., 2017 [124]	Fingolimod	EAE	Combined administration of fingolimod and NSCs ameliorated clinical signs and CNS demyelination, promoted remyelination, prevented neurodegeneration and astrogliosis
Rothhammer et al., 2017 [128]	Fingolimod	EAE in NOD mice	Reduced CNS pathogenic innate immune activation
Gentile et al., 2016 [129]	Siponimod	EAE	Improved EAE clinical scores, attenuation of astrogliosis and microgliosis, prevented loss of striatal GABAergic neurons, reduced lymphocyte infiltration in striatum
Tiwari-Woodruff et al., 2016 [135]	Siponimod	Cuprizone mouse model	Prevents neurodegeneration and demyelination
Hundehege et al., 2019 [131]	Siponimod	Focal EAE	Partial restoration of neuronal network integrity
Ward et al., 2020 [130]	Siponimod	EAE	Reduced production of TH17 by T cells, diminished subpial demyelinating lesions
Scott et al., 2016 [136]	Ozanimod	EAE	Dose-dependent amelioration in clinical severity of EAE, transient peripheral lymphopenia
Musella et al., 2020 [134]	Ozanimod	EAE	Partial restore of striatal glutamatergic dysfunction caused by microglia/macrophage activation
Hou et al., 2021 [137]	Ponesimod	EAE	Restores the Th1/Th17/Treg balance and ameliorates disease severity
Komiya et al., 2013 [138]	ONO-4641 (Ceralifimod)	EAE in NOD mice	Prevents relapses of relapsing−remitting EAE, dose-dependent blockage of lymphocyte infiltration in CNS

EAE: experimental autoimmune encephalomyelitis, TNF-α: tumor necrosis factor-α, IL1β: interleukin 1β, IL6: interleukin 6, OPC: oligodendrocyte progenitor cell, NSC: neural stem cell, NOD: nonobese diabetic mice.

**Table 3:** S1PR modulators in the treatment of MS and IBD.

Name	S1PR Subtype Modulation	Disease	Indication	Approval Phase
Fingolimod (FTY720)	S1PR1,3,4,5	MS ^1^	Relapsing forms of MS	Approved [149]
Siponimod (BAF312)	S1PR1,5	MS	Relapsing forms of MS	Approved [150]
Ponesimod (ACT128800)	S1PR1	MS	Relapsing forms of MS	Approved [151]
Ozanimod (RPC1063)	S1PR1,5	MS IBD ^2^	Relapsing forms of MS Moderate to severe UC ^3^ Moderate to severe CD ^4^	Approved [152] Phase III (NCT02531126, NCT03915769) PhaseIII (NCT03467958, NCT03440385, NCT03440372, NCT03464097)
Amiselimod (MT-1303)	S1PR1,4,5	MS IBD	RRMS Moderate to severe CD Mild to moderate UC	Phase II (completed) [153,154] Phase II (completed) (NCT02389790) Phase II (NCT04857112)
Etrasimod (APD334)	S1PR1,4,5	IBD	Moderate to severe UC Moderate to severe CD	Phase III (NCT03950232) Phase IIb (NCT04173273)

^1^ RRMS: relapsing−remitting multiple sclerosis, ^2^ IBD: inflammatory bowel disease, ^3^ UC: ulcerative colitis, ^4^ CD: Crohn’s disease.
